# Supplementary material for: Netcombin: An algorithm for constructing optimal phylogenetic network from rooted triplets
Source: PLoS One. 2020 Sep 18;15(9):e0227842. doi: 10.1371/journal.pone.0227842 (PMC7500971; doi:10.1371/journal.pone.0227842)
Supplement: S4 File — (ZIP) [file pone.0227842.s004.zip › SequenceData-Triplet/sequenceData-10-20-30-0-Triplet/length 200, numseq 40, rate 10^-7/9 seq.pdf]

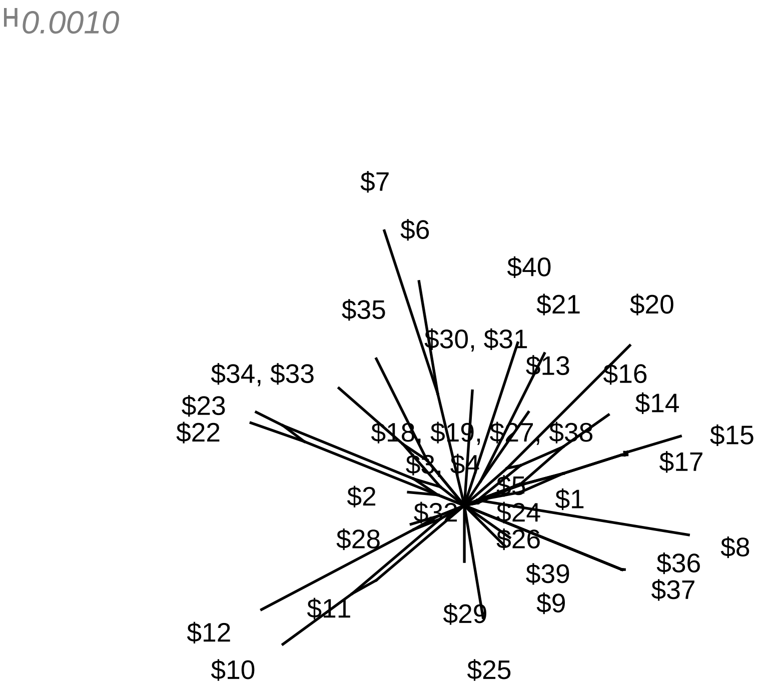
0.0010
$7
$6
$40
$35
$21
$20
$30, $31
$34, $33
$13
$16
$23
$14
$22
$18, $19, $27, $38
$15
$17
$3, $4
$5
$2
$1
$32
$24
$28
$26
$8
$36
$39
$37
$9
$11
$29
$12
$10
$25
